# The Clinical Features of Children With Acute Fulminant Myocarditis and the Diagnostic and Follow-Up Value of Cardiovascular Magnetic Resonance

**DOI:** 10.3389/fped.2019.00388

**Published:** 2019-10-01

**Authors:** Jianli Lv, Bo Han, Cuiyan Wang, Jing Wang, Diandong Jiang, Lijian Zhao, Yingchun Yi, Jianjun Zhang

**Affiliations:** ^1^Department of Pediatric Cardiology, Shandong Provincial Hospital Affiliated to Shandong University, Jinan, China; ^2^Shandong Medical Imaging Research Institute, Jinan, China

**Keywords:** children, acute fulminant myocarditis, cardiovascular magnetic resonance, pacemaker, ECMO

## Abstract

**Objective:** To investigate the clinical features and the diagnostic and follow-up value of acute fulminant myocarditis (AFM) in children.

**Methods:** A total of 20 children diagnosed with AFM admitted to our department were reviewed, and the clinical manifestations; pathogenic examination results; myocardial injury biomarkers; and electrocardiography, echocardiogram, and cardiovascular magnetic resonance (CMR) results were analyzed.

**Results:** Twenty children with AFM, including 12 males and 8 females, aged 3–16 years, were analyzed. The initial symptoms were abdominal pain, vomiting, fatigue, syncope, and convulsions. All children had significantly increased hs-cTnT and NT-pro BNP. In addition to nonspecific ST-T changes, there were 10 cases of complete atrioventricular block, 2 cases of advanced atrioventricular block, and 1 case of ventricular tachycardia. Echocardiography showed an increase in the cardiac chamber sizes in 15 patients and a decrease in left ventricular ejection fraction (LVEF) in 17 patients. There were 16 patients with abnormal CMR findings, including 13 cases of high T2-weighted image (T2WI) signal and 14 cases of late gadolinium enhancement (LGE). In the patients who underwent CMR within 14 days of onset, the sensitivity of T2WI and LGE and the positive diagnosis rate were higher than in those who underwent CMR after 14 days, but the difference was not statistically significant. CMR was followed up in 10 patients: 7 patients returned to normal, 2 patients still had mild LGE, and 1 patient developed inflammatory dilated cardiomyopathy. All patients were treated with high-dose immunoglobulin, 11 of whom received high-dose immunoglobulin combined with glucocorticoids. Eight patients received temporary pacemakers, and 1 patient received ECMO. None of the patients died. The peak of hs-cTnT was significantly higher in the glucocorticoid group than in the unused glucocorticoid group (2853.4 ± 2217.2 and 1124.7 ± 527.3 pg/ml, respectively).

**Conclusion:** Children with AFM have unique clinical features. Early identification and effective treatment can reduce the mortality rate and improve the prognosis. CMR is highly sensitive in the diagnosis of ARM, especially within 14 days of onset, and is a useful noninvasive imaging technique for the early identification of AFM in children. The dynamic observation and follow-up of children with AFM through CMR can guide clinical decision-making and prognosis assessment.

## Introduction

Acute fulminant myocarditis (AFM) in children has a rapid onset and progresses rapidly. Most of the onsets are concealed. Extracardiac manifestations are predominant. It is difficult to make an early diagnosis. Various arrhythmias, Adams-Stokes syndrome, heart failure, cardiogenic shock, and even sudden cardiac death can occur within 24–48 h of onset. AFM is a clinically critical pediatric illness with a high mortality rate. A recent national survey in Japan found that the survival rate of children with AFM was only 48.6% (1). The key to affecting the mortality rate is related to early identification and effective treatment. Cardiovascular magnetic resonance (CMR) has received increasing attention in the diagnosis of children with AFM. It has been reported in the literature that the positive predictive value of CMR for acute myocarditis is over 90%. However, for patients with AFM, due to the serious condition and the inconvenience of the examination, the clinical application of CMR is greatly limited. Our early studies have shown that CMR is more sensitive than AFM in the diagnosis of conventional myocarditis. This study retrospectively analyzed the clinical data and CMR findings of 20 children with AFM and explored the clinical features of AFM in children and the value of CMR in diagnosis, treatment, and prognosis to provide valuable and important information for the diagnosis and treatment of this disease.

## Materials and Methods

### Research Subjects

Twenty patients with AFM admitted to our hospital from November 2011 to March 2019 were enrolled, including 12 males and 8 females aged 3–16 years (8.4 ± 3.1 years).

### Ethics Statements

This study was carried out in accordance with the recommendations of the Ethics Committee of Shandong Provincial Hospital Affiliated to Shandong University. All subjects gave written informed consent in accordance with the Declaration of Helsinki. The protocol was approved by the Ethics Committee of Shandong Provincial Hospital Affiliated with Shandong University.

The clinical data of the children were reviewed, including clinical manifestations; pathogenic examination results; myocardial injury biomarkers; electrocardiogram, echocardiography, and cardiac MRI results; treatment methods; and outcomes.

Inclusion criteria: ① a diagnosis of acute myocarditis in line with the criteria for the clinical diagnosis of myocarditis in the *Diagnostic Recommendations for Children with Myocarditis* (2018 edition) published by the Subspecialty Group of Cardiology of the Society of Pediatrics of Chinese Medical Association (2); and ② a diagnosis of AFM, which refers to clinical manifestations of severe heart failure within 2 weeks of onset (cardiac function level IV) and acute myocarditis requiring positive inotropic drugs, vasopressors, and/or mechanical circulation support to maintain heart function or blood pressure (3).

Exclusion criteria: nonischemic cardiomyopathy, congenital heart disease, myocardial infarctions and other diseases that can explain the clinical manifestations.

### CMR

The machine used for the inspection was the 3.0T Skyra from Siemens. The heart rate is required to be 120 beats/min or less during the examination. The scan sequence includes gradient echo sequence, spin echo sequence, and inversion recovery fast spin echo sequence, first perfusion scan and late gadolinium enhancement (LGE). The contrast agent used in LGE was gadolinium-diethylenetriaminepentacetate (Gd-DTPA).

### CMR Criteria for the Diagnosis of Myocarditis (4)

The diagnosis is established when the CMR performance meets two or more of the following three criteria: ① Regional or global myocardial signal intensity increases in T2-weighted images (T2WI); ② Increased global myocardial early enhancement ratio between the myocardium and skeletal muscle in gadolinium-enhanced T1-weighted images (T1WI); There is at least 1 focal lesion with nonischemic regional distribution in inversion recovery-prepared late gadolinium-enhanced T1WI (LGE).

### Statistical Analysis

SPSS 25.0 statistical software was used, and the measured data are expressed as the range (mean ± standard deviation). The *t*-test was used to compare the two samples. Correlation analysis was performed with the Pearson correlation coefficient test. The count data are expressed by frequency (rate) and were analyzed by Fisher's exact probability method using the χ^2^ test. *P* < 0.05 was considered statistically significant.

## Results

The main clinical data of 20 children with AFM are shown in Table 1.

**Table 1:** Main clinical data of 20 children with AFM.

**Patient #**	**Age (yr)**	**Gender**	**Symptoms**	**Pathogens**	**hs-cTnT (pg/ml)**	**ECG**	**ECHO**		**CMR**			**Treatment**		**LOS**
							**CCE**	**LVEF (%)**	**FIT**	**T2WI**	**LGE**	**UOG**	**MCS**	
1	9	F	Abdominal pain, vomiting	ASO	2,102	–	Yes	25	7	–	+	Yes	–	34
2	6	M	Vomiting	MP	1,632	CAVB	Yes	40	11	+	+	Yes	–	28
3	13	M	Syncope	–	1,967	CAVB	Yes	38	12	+	+	Yes	Pacemaker	33
4	7	M	Abdominal pain	–	2,783	CAVB	Yes	60	17	–	–	Yes	Pacemaker	16
5	8	F	Abdominal pain, vomiting	MP	6,457	–	Yes	33	13	+	+	Yes	–	29
6	10	F	Abdominal pain	ASO	269.2	–	Yes	30	24	–	–	Yes	–	32
7	12	F	Abdominal pain	–	1,791	CAVB	Yes	42	13	–	–	Yes	–	25
8	3	M	Abdominal pain, vomiting	–	1,864	AAVB	Yes	48	12	+	+	Yes	–	24
9	8	M	Convulsion	MP	956	CAVB	No	41	17	+	+	No	Pacemaker	22
10	16	F	Chest tightness	EBV	3,367	–	No	43	11	+	+	Yes	–	28
11	10	M	Fatigue	HHV6	1,550	CAVB	No	61	69	–	–	Yes	Pacemaker	34
12	9	M	Vomiting	MP	822.3	–	Yes	38	6	+	–	No	–	13
13	7	M	Palpitation	EBV, HHV6	212	–	Yes	37	20	+	+	No	–	26
14	5	M	Vomiting	MP	1,011	AAVB	Yes	50	12	-	+	No	–	19
15	10	F	Fatigue	–	1,164	–	Yes	35	10	+	+	No	–	22
16	9	F	Syncope	MP	1,477	CAVB	No	62	10	+	+	No	Pacemaker	23
17	7	F	Vomiting	ASO	1,105	CAVB	Yes	39	16	+	+	No	Pacemaker	19
18	3	M	Fatigue	–	1,188	CAVB	Yes	37	11	+	+	No	Pacemaker	31
19	7	M	Abdominal pain, vomiting	–	7,605	VT	Yes	20	23	+	+	Yes	ECMO	26
20	9	M	Vomiting	–	2,187	CAVB	No	45	38	–	+	No	Pacemaker	42

*hs-cTnT, hypersensitive cardiac troponin T; CCE, cardiac chamber enlargement; LVEF, left ventricular ejection fraction; FIT, first inspection time; UOG, use of glucocorticoid; MCS, mechanical circulation support; LOS, length of stay; ASO, anti-streptolysin O; MP, mycoplasma pneumonia; EBV, Epstein-Barr virus; HHV6, human herpesvirus 6; CAVB, complete atrioventricular block; AAVB, advanced atrioventricular block; VT, ventricular tachycardia*.

### Initial Symptoms

The initial symptoms were abdominal pain and vomiting in 12 patients (60%), chest tightness and fatigue in 4 patients (20%), syncope and convulsion in 3 patients (15%), and palpitation in 1 patient (5%). The time from onset to admission was 0–7 days (3 ± 1.6 days). The most serious patients were often hospitalized within a few hours because of the deterioration of their condition, which manifested as heart failure, cardiogenic shock, Adams-Stokes syndrome, or severe arrhythmia.

### Pathogen Examination

All patients underwent pathogen examinations after admission, including EBV, cytomegalovirus, coxsackie virus, parvovirus B19, adenovirus, HHV6, and hepatitis virus detection; influenza A and B virus nucleic acid detection; MP-IgM antibody and ASO detection; and sputum and blood bacterial culture analysis. There were a total of 12 abnormalities, including 6 patients positive for MP-IgM, 3 patients positive for ASO, 1 patient with EBV infection, 1 patient with HHV6 infection, and patient with mixed EBV and HHV6 infection.

### hs-cTnT and NT-Pro BNP Results

**Figure 1:**
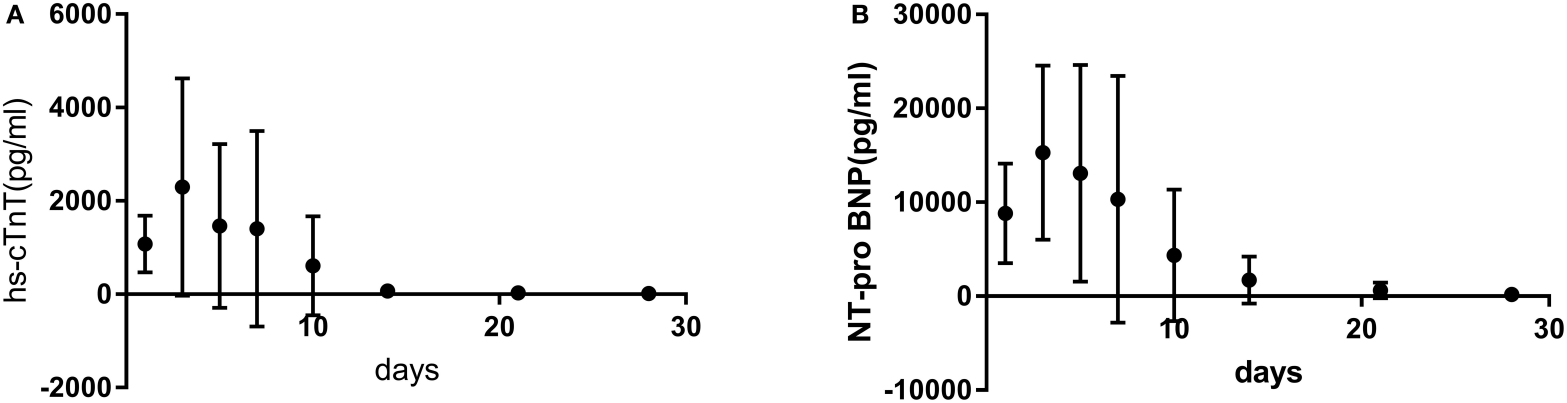
The trend of hs-cTnT **(A)** and NT-pro BNP **(B)** with the course of disease.

hs-cTnT and NT-pro BNP were significantly increased in 20 children with AFM. Both peaked at 3–7 days (3.9 ± 1.0 days) after onset, then gradually decreased, and after 14–30 days (21.6 ± 5.5 days) returned to normal (Figure 1). Pearson correlation analysis confirmed that there was a significant correlation between hs-cTnT and NT-pro BNP (correlation coefficient 0.788–0.921, *P* < 0.05).

### Electrocardiogram

Twenty patients underwent routine 12-lead ECG after admission. In addition to nonspecific ST-T abnormalities, there were 10 (50%) patients with CAVB, 2 (10%) with AAVB, and 1 (5%) with VT. The conduction block occurred within 0–4 days (1.2 ± 1.3 days) after onset, and conduction was mostly restored after 1–9 days (3.6 ± 2.5 days). However, there was still one patient with first-degree atrioventricular block combined with complete right bundle branch block during follow-up.

### Echocardiography

Twenty patients were admitted to the hospital for echocardiography, and 18 patients (90%) had positive results for AFM. The diameter of the heart chamber was increased in 15 patients (75%), with 9 cases involving the left heart, 1 case involving the right heart, and 5 cases involving the whole heart. Except for 2 patients that did not return to normal, the remaining 13 patients returned to normal after 10–26 days (16.9 ± 4.9 days). Seventeen patients (85%) had a decreased LVEF (<60%) with an EF value of 20–50% (37.7 ± 7.7%):14 patients returned to normal within 30 days (16.4 ± 4.4 days), 2 patients returned to normal within 3 months, and 1 patient did not recover for half a year. Electrocardiography results from 10 patients (50%) showed a thickening of the ventricular wall that mainly involved the left ventricle. All patients had a reduction in wall motion, mainly in the interventricular septum and left ventricular inferior wall. There were 13 patients (65%) with pericardial effusion, mostly light, and only 1 case was moderate. Mitral regurgitation occurred in 17 patients (85%), mostly small or moderate. Myocardial thinning occurred in 4 patients, 3 of whom had thinning in the apex of the anterior wall and 1 in the ventricular septum.

### CMR

#### Time and Safety of CMR Imaging

The 20 children with AFM were divided into group A (acute stage, onset within 14 days) and group B (recovery stage, onset after 14 days) according to the time of the first CMR examination. There were 12 patients (60%) in group A and 8 patients (40%) in group B. The examination time of group A was 6–13 days (10.7 ± 2.2 days) after onset and that of group B was 16–69 days (28.0 ± 18.0 days). Ten patients were followed up for 25–144 days (69.0 ± 38.6 days).

All the children successfully completed the CMR examination, and none of them had contrast agent allergy or other complications. Each examination took approximately 40 min to 1 h.

#### Total Inspection Results of Groups A and B

There were 16 patients (80%) with abnormal CMR imaging out of the 20 children with AFM. The main results were 13 cases (65%) of high T2WI signal, 14 cases (70%) of LGE, 5 cases of myocardial thinning, 6 cases of myocardial motility reduction, 6 cases of pericardial effusion, and 3 cases of myocardial perfusion defect.

### CMR Results of Group A

Ten of the 12 patients in the acute phase who underwent CMR in the acute phase had abnormal results. There were 9 cases (75%) of high T2WI signal, including 7 cases in the ventricular septum and 2 cases in the left ventricle. LGE was found in 9 patients (75%), including in the ventricular septum in 2 patients, in the left ventricle in 5 patients, and in both the ventricular septum and left ventricle in 2 patients. The myocardium was thinned in 3 patients, including in the left ventricular wall in 2 patients and in the apex in 1 patient. Myocardial mobility was reduced in 4 patients. There were 5 cases of pericardial effusion, all of which were light.

Eight patients in group A underwent CMR follow-up, of whom 6 returned to normal after 25–63 days of onset, and 2 patients still had mild LGE at 33 and 46 days of onset, respectively, although the lesion range was smaller than before.

### CMR Results of Group B

Six of the 8 patients in group B who underwent CMR during the recovery period had abnormal results. There were 4 cases (50%) of high T2WI signal, including 3 cases in the ventricular septum and 1 case in the left ventricle. LGE was found in 5 patients (62.5%), including in the ventricular septum in 3 patients and in both the ventricular septum and left ventricle in 2 patients. There were 3 cases of perfusion defects, including 1 case in the ventricular septum and 2 cases in the apex. Myocardial thinning occurred in 2 patients, including in the ventricular septum in 1 patient and in both the ventricular septum and left ventricular inferior wall in 1 patient. Myocardial mobility was reduced in 2 patients. Pericardial effusion occurred in one patient.

Two patients in group B underwent CMR follow-up. One of the patients had no abnormalities in the initial examination and follow-up examination. The other patient had significant cardiac enlargement, LVEF reduction, periventricular septal defect and LGE at 129 days of onset and was diagnosed with inflammatory dilated cardiomyopathy.

### Comparison of T2WI, LGE Sensitivity, and Diagnostic Positive Rates Between Group A and B

There were 9 cases of T2WI high signal in the 12 patients of group A and 4 cases of T2WI high signal in the 8 patients of group B. There was no significant difference between the two groups (*P* > 0.05). There were 9 cases of LGE in the 12 patients of group A and 5 cases of LGE in the 8 patients of group B. There was also no significant difference between the two groups (*P* > 0.05). According to the Lake Louise criteria, 8 patients (66.7%) in group A were diagnosed with myocarditis (both T2WI high signal and LGE), and there were 4 patients with myocarditis (50%) in group B. There was no significant difference between the two groups (*P* > 0.05).

### Treatment and Outcome

Twenty patients were treated with bed rest; oxygen; and anti-infection, myocardial nutrition, anti-heart failure, anti-arrhythmia, and other comprehensive treatments after admission. All patients were treated with high-dose human immunoglobulin (IVIG, total 2 g/kg, divided into 2–5 days). We used glucocorticoids in 11 patients who had no improvement in heart failure or a persistent increase in hs-cTnT after 2–3 days of routine treatment. The dosage of glucocorticoids was generally 1–2 mg/kg/d of prednisone, which was gradually decreased according to the condition of the patients. Six of these patients received an impact dose, methylprednisolone 10–30 mg/kg/d (total amount not exceeding 1 g), for 3 days. The total course of glucocorticoid treatment was 6–46 days (26.1 ± 12.9 days). The hs-cTnT peak in children in the glucocorticoid group was higher than that in the children in the unused glucocorticoid group (2853.4 ± 2217.2 and 1124.7 ± 527.3 pg/ml, respectively), and the difference was statistically significant (*P* < 0.05; Table 2). There was no significant difference between the length of stay (28.1 ± 5.4 vs. 24.1 ± 8.3 days) and the normal time of LVEF recovery (16.9 ± 5.2 vs. 27.3 ± 25.0 days) (*P* > 0.05) in the glucocorticoid group vs. the unused glucocorticoid group (Table 2). Eight patients were treated with a temporary pacemaker for 1–33 days (9.1 ± 10.2 days), and one patient was treated with ECMO for 4 days. All children were discharged, and no child died. In group A, in 2 patients with mild LGE during their CMR review, their cardiac size and function returned to normal after treatment with myocardial nutrition, beta-blocker, ACEI, aldosterone antagonist, or other drug therapy. Children who were treated with ECMO in group B did not recover at 5 months after treatment with the above method and were still in follow-up.

**Table 2:** Comparison of the hs-cTnT peak and the length of stay between the glucocorticoid group and the unused glucocorticoid group.

	**Glucocorticoid group (*n* = 11)**	**Unused glucocorticoid group (*n* = 9)**	***P***
hs-cTnT peak (pg/ml)	2853.4 ± 2217.2	1124.7 ± 527.3	<0.05
Time of hs-cTnT to normal (days)	21.6 ± 5.9	22.0 ± 5.7	>0.05
Time of LVEF to normal (days)	16.9 ± 5.2	27.3 ± 25.0 (*n* = 8)	>0.05
Length of stay(days)	28.1 ± 5.4	24.1 ± 8.3	>0.05

## Discussion

AFM is a cardiovascular crisis in children, and the incidence rate is increasing. In 1991, Lieberman et al. classified myocarditis into four types, fulminant, acute, chronic, and chronic, based on histological changes and the clinical manifestations of myocardial biopsy (5). In 2013, Ginsberg et al. defined AFM as acute myocarditis with clinical manifestations of sudden onset with severe hemodynamic disorders. Clinical symptoms markedly and rapidly worsen, requiring positive inotropic drugs, mechanical ventilation, or mechanical circulation aids to provide hemodynamic support (3). At present, the gold standard for AFM diagnosis is still endomyocardial biopsy (EMB), but because of its invasiveness and low sensitivity, it is not used as a first-line examination, especially for children (4, 6). Currently, the diagnosis of AFM mainly depends on the clinical manifestations; cardiac injury biomarkers; and electrocardiogram, chest X-ray and echocardiography results (7, 8). Therefore, this study chose to meet Ginsberg's definition of AFM as the research objective.

The etiology and mechanism of this disease are unknown. Viral infection, autoimmune disease, drug poisoning, giant cell myocarditis, and sarcoidosis can induce AFM. Viral infection is the most common and difficult to foresee. There are a wide variety of pathogenic viruses, but due to the limitations of detection methods, only 10–20% of patients with acute myocarditis demonstrate the presence of viral genes in myocardial tissue upon detection. Myocarditis is usually caused by viral infections and postviral immune-mediated responses. With the development of new molecular technologies, such as polymerase chain reaction (PCR) and *in situ* hybridization, the most frequently detected viral profiles in EMB have changed from typical enteroviruses and adenoviruses to the major parvoviruses B19 (PVB19) and HHV6 (9, 10). In recent years, influenza viruses, especially influenza A viruses, are more common (11, 12). In addition, myocarditis can be caused by nonviral infections such as parasites, MP, *Streptococcus pneumoniae*, etc. (13–15). Mahfoud et al. studied the diagnostic value of viral serology and EMB viral genome detection in patients with clinically suspected myocarditis. Only 5 (4%) of 124 patients had serological evidence, and the same viral infection was detected by PCR in EMB samples (16). This result indicates that viral serology is not yet suitable for the diagnosis of myocardial infection in patients with suspected myocarditis. In our study, the pathogen examination was more common with MP-IgM and ASO. However, because EMB was not performed, we were unable to determine its relevance to AFM. However, clinicians are reminded to be alert to the possible pathogenesis of MP and *Streptococcus pneumonia* infection.

The initial symptoms of AFM often do not manifest as cardiovascular symptoms but as atypical symptoms such as vomiting, abdominal pain, cough, and syncope, and the clinical misdiagnosis rate is high. In this study, the initial symptoms of the digestive system, such as abdominal pain and vomiting, occurred in 60% of patients, respiratory symptoms such as chest tightness and fatigue occurred in 20%, and heart and other related symptoms occurred in only 5%. In clinical work, in patients with abdominal pain and chest tightness, especially in those with prodromal symptoms such as fever and cough, with hemodynamic disorder or malignant arrhythmia as the main manifestation, doctors should focus on whether the patient has AFM.

At present, the commonly used myocardial injury biomarkers are the creatine kinase isoenzyme and cardiac troponin (cTn). In the early stage of AFM, cTn increased more frequently than the creatine kinase isoenzyme (17), and the degree of cTn increase can help to judge the prognosis. cTn is a nonenzymatic serum marker with high specificity (90%) and high sensitivity for evaluating myocardial injury, and it is the preferred marker for detecting myocardial injury (18). cTn usually rises within 2–4 h of onset and falls to normal for 2–3 weeks. The hs-cTnT level in this group reached a peak at 3–5 days after onset and rapidly decreased to normal after the disease improved, which was consistent with the above experience.

Typical manifestations of main electrocardiograms in the acute phase of AFM include extensive ST-T changes with atrioventricular block and various ectopic arrhythmias. A continuous prolongation of the QRS interval (>120 ms) is considered an independent predictor of cardiogenic death or heart transplantation (8). The most common atrioventricular block in this group was CAVB, most of which occurred within 3–5 days of onset, with an average return to normal within 3.6 ± 2.5 days. There was one case of continuous QRS interval (≥120 ms) that eventually developed into inflammatory dilated cardiomyopathy.

Echocardiography can assess ventricular size, wall thickness, the amplitude of motion, systolic and diastolic function, pericardial effusion, and endoluminal thrombosis in patients with AFM. Its most important role is to rule out other causes of heart failure, such as valvular heart disease or other cardiomyopathy (hypertrophic or restrictive cardiomyopathy) (19). Many previous studies have reported that the heart chamber size of patients with AFM is normal, and these patients mainly show an increase in the interval thickness due to acute myocardial edema, whereas the heart chamber of patients with common myocarditis is enlarged, and this helps to distinguish between AFM and common myocarditis (3, 20). The above studies are all results of adult AFM. In contrast, this group of children with AFM showed an increase in the diameter of the heart cavity in 75% of the patients, mainly a mild expansion. We consider that the reasons may be related to the following aspects: (1) the heart during childhood is still in the process of development, and the myocardial compliance is poor and cannot tolerate increased preload; (2) the sympathetic distribution of the myocardium is not mature enough to release enough catecholamine to regulate the response of the myocardium to the increased preload; (3) the conduction system of children with AFM is more susceptible, and atrioventricular block slows the heart rate and reduces cardiac output.

CMR is gaining increasing attention in the diagnosis of myocarditis. It can detect the location, extent and degree of myocardial injury, which cannot be detected by echocardiography. Abdel-Aty et al. reported that the sensitivity, specificity, and accuracy of T2WI in the diagnosis of myocarditis were 84, 74, and 79%, respectively; those of early enhancements were 80, 68, and 74.5%, respectively; and those of LGE were 44, 100, and 71%, respectively. Optimal diagnostic performance was produced when “any two” of the three sequences were positive in the same patient, with 76% sensitivity, 95.5% specificity, and 85% diagnostic accuracy (21). However, for patients with AFM, their options for clinical examination are greatly limited due to their poor condition and the inconvenience of the examination. We used CMR to examine the 20 patients with AFM, and the sensitivity of T2WI was 65% and that of LGE was 75%. Due to the criticality of the condition, CMR was performed only when the patient's hemodynamics were stable. Therefore, 20 patients had different examination times. We found that the sensitivity of CMR was different between the acute phase and the recovery phase: T2WI sensitivity was 75 and 50%, respectively, in the acute phase and the recovery phase, and LGE sensitivity was 75 and 62.5%, respectively, in the acute phase and the recovery phase, suggesting that myocardial edema began to resolve 14 days after onset while LGE was relatively delayed. In 2016, Wang et al. performed an early CMR examination and short-term follow-up of 8 children with AFM (22). It was found that CMR has certain value for the early diagnosis and short-term follow-up of children with AFM. Through the study of more patients and longer follow-up, we found that the sensitivity of CMR examination within 14 days was higher, and the sensitivity decreased significantly over 14 days. Therefore, we suggest that CMR should be performed as soon as possible if the patient's condition permits. In addition, we compared the results of echocardiography with those of CMR during follow-up, and these results confirmed that CMR could show abnormalities that could not be recognized by echocardiography and further confirmed that CMR had important value in the early diagnosis and long-term evaluation of children with AFM.

In terms of treatment, in recent years, an increasing number of small-scale studies and case reports have suggested that IVIG is beneficial for patients with AFM and inflammatory dilated cardiomyopathy and can improve LVEF and long-term prognosis with no obvious adverse reactions (14, 23). We performed IVIG treatment on this group of 20 children with AFM, and it also proved beneficial to these patients. Chen et al. conducted a controlled study of 719 patients with myocarditis and found that glucocorticoids may improve cardiac function but did not reduce mortality (24). In a long-term follow-up study (mean follow-up of 33 months), 21% of patients with myocarditis developed dilated cardiomyopathy (25). Studies by Kuhl et al. show that the persistence of the viral genome leads to chronic inflammation, which affects the recovery of LVEF (26) and may be related to progression to inflammatory dilated cardiomyopathy. However, some case reports and small-scale clinical studies have shown the beneficial effects of glucocorticoid therapy on AFM (27, 28). Therefore, the therapeutic effect of glucocorticoids on children with AFM is still controversial. Ginsberg et al. believe that immunosuppressive therapy should not be routinely used to treat myocarditis, but it is strongly recommended to use it after conventional anti-heart failure treatment (3). In this study, 11 patients with severe condition (mean hs-cTnT average 2853.4 ± 2217.2 pg/ml) were treated with IVIG and glucocorticoid therapy. The comparison of hospitalization time with children without glucocorticoids proved that glucocorticoids can improve children's condition but do not affect the prognosis. In theory, the use of immunosuppressive agents in the acute phase of viral replication may aggravate the direct damage of the virus to the myocardium, and the use of immunosuppressive agents during the acute phase of immune-mediated injury may bring some benefits, but how to grasp the timing still requires more research. In addition, 8 patients in this study received temporary pacemakers, and 1 patient received ECMO to ensure effective tissue perfusion. Therefore, we recommend that patients with AFM with CAVB should receive temporary pacemakers in a timely manner, and those who cannot maintain the circulation stability for conventional treatment methods should receive ECMO and other mechanical circulation aids in time, which can play a crucial role in improving the prognosis of patients with AFM.

The limitations of this study are that the sample size is small and it is a retrospective clinical study, the accuracy and integrity of which cannot be guaranteed. AFM was only clinically diagnosed, EMB was not performed, and histopathological diagnosis and virus isolation were not used to determine the cause. In addition, although studies have shown that CMR has a high sensitivity for the diagnosis of AFM, it is necessary to conduct a randomized prospective trial to demonstrate its specificity in the diagnosis of these patients.

## Conclusion

AFM has a rapid onset and can progress to heart failure and cardiogenic shock in a short period of time. The clinical presentation of symptoms of AFM in children are more commonly in the gastrointestinal and respiratory systems, and AFM is difficult to diagnose early and is easily misdiagnosed. CMR is highly sensitive for the diagnosis of AFM, especially within 14 days of onset. It can show not only the location of myocardial injury but also the degree and extent of myocardial inflammation, as well as the repair of fibrosis after inflammation, which is helpful for the early identification of children with AFM. The dynamic observation and follow-up of the course of AFM in children by cardiac MRI can be used to guide clinical decision-making and evaluate prognosis. The development of life support technologies such as emergency cardiac pacing and ECMO has reduced the mortality rate of children with AFM.

## Data Availability Statement

The raw data supporting the conclusions of this manuscript will be made available by the authors, without undue reservation, to any qualified researcher.

## Ethics Statement

Written informed consent was obtained from the minor(s)' legal guardian/next of kin for the publication of any potentially identifiable images or data included in this article.

## Author Contributions

JL performed original literature search, developed methodology, performed statistics, summary and analysis of data, and prepared first draft of manuscript and revision. BH conceived the idea, performed original literature search, organized and coordinated the investigation, managed patients, and performed data analysis and manuscript revision. CW performed CMR and analyzed CMR data. JW performed literature search, data collection and data analysis, and provided critical advice on the manuscript. DJ managed patients and advised on data analysis and the manuscript. LZ, YY, and JZ managed patients and provided critical revision and advice on the research protocol and manuscript. All authors approved this version of the manuscript to be published and agreed to be accountable for all aspects of the work, thereby ensuring that questions related to the accuracy or integrity of any part of the work are appropriately investigated and resolved.

### Conflict of Interest

The authors declare that the research was conducted in the absence of any commercial or financial relationships that could be construed as a potential conflict of interest.
